# A meta-analysis of the diagnostic performance of machine learning-based MRI in the prediction of axillary lymph node metastasis in breast cancer patients

**DOI:** 10.1186/s13244-021-01034-1

**Published:** 2021-11-03

**Authors:** Chen Chen, Yuhui Qin, Haotian Chen, Dongyong Zhu, Fabao Gao, Xiaoyue Zhou

**Affiliations:** 1 Department of Radiology, West China Hospital, Sichuan University, 37 Guoxue Road, Chengdu, 610041 Sichuan People’s Republic of China; 2 Siemens Healthineers Ltd., Shanghai, People’s Republic of China

**Keywords:** Artificial intelligence, Axillary lymph node metastasis, Machine learning, Magnetic resonance imaging

## Abstract

**Background:**

Despite that machine learning (ML)-based MRI has been evaluated for diagnosis of axillary lymph node metastasis (ALNM) in breast cancer patients, diagnostic values they showed have been variable. In this study, we aimed to assess the use of ML to classify ALNM on MRI and to identify potential covariates that might influence the diagnostic performance of ML.

**Methods:**

A systematic research of PubMed, Embase, Web of Science, and the Cochrane Library was conducted until 27 December 2020 to collect the included articles. Subgroup analysis was also performed.

**Findings:**

Fourteen studies assessing a total of 2247 breast cancer patients were included in the analysis. The overall AUC for ML in the validation set was 0.80 (95% confidence interval [CI] 0.76–0.83) with a negative predictive value of 0.83. The pooled sensitivity and specificity were 0.79 (95% CI 0.74–0.84) and 0.77 (95% CI 0.73–0.81), respectively. In the subgroup analysis of the validation set, T1-weighted contrast-enhanced (T1CE) imaging with ML yielded a higher sensitivity (0.80 vs. 0.67 vs. 0.76) than the T2-weighted fat-suppressed (T2-FS) imaging and diffusion-weighted imaging (DWI). Support vector machines (SVMs) had a higher specificity than linear regression (LR) and linear discriminant analysis (LDA) (0.79 vs. 0.78 vs. 0.75), whereas LDA showed a higher sensitivity than LR and SVM (0.83 vs. 0.70 vs. 0.77).

**Interpretation:**

MRI sequences and algorithms were the main factors that affect the diagnostic performance of ML. Although its results were encouraging with the pooled sensitivity of around 0.80, it meant that 1 in 5 women that would go with undetected metastases, which may have a detrimental effect on the overall survival for 20% of patients with positive SLN status. Despite that a high NPV of 0.83 meant that ML could potentially benefit those with negative SLN, it might also translate to 1 in 5 tests being false negative. We would like to suggest that ML may not be yet usable in clinical routine especially when patient survival is used as a primary measurement of its outcome.

**Supplementary Information:**

The online version contains supplementary material available at 10.1186/s13244-021-01034-1.

## Key points

- Sentinel lymph node biopsy (SLNB) with machine learning (ML) might be more helpful to breast cancer patients because ML might prevent over-treatment due to its sensitivity of around 0.80 to classify ALNM.
- T1CE with ML is more sensitive than T2-FS and DWI (0.80 vs. 0.67 vs. 0.76, respectively).
- Support vector machine (SVM) is more specific than linear regression (LR) and linear discriminant analysis (LDA) (0.79 vs. 0.78 vs. 0.75, respectively), whereas LDA is more sensitive than LR and SVM (0.83 vs. 0.70 vs.0.77, respectively).

## Introduction

Breast cancer is one of the most common malignancies worldwide, accounting for 30% of all new cancer diagnoses in 2018 among American women [1]. As axillary lymph node status in breast cancer patients is crucial for pathologic staging, it is also used as a prognostic indicator and for clinical patient management, therapeutic guidance, and survival predictions [2, 3]. Although axillary lymph node dissection (ALND) is the gold standard for evaluating axillary lymph node metastasis (ALNM), ALND might not confer a survival advantage [4]. Sentinel lymph node biopsies (SLNBs) are used widely and can reduce ALND complications [5]. However, SLNBs are invasive procedures that could be associated with fewer disadvantages such as lymphoedema and sensory loss (the risks of 5% and 11%, respectively) [6]. One way of SLNBs is to surgically remove of one or a few axillary lymph nodes, whereas over 70% of SLNBs are negative, thus questioning the generic use of this invasive procedure [7]. In addition, another way of SLNBs is to inject a radiotracer or ultrasound with fine-needle aspiration, which, however, is difficult to perform in primary hospitals due to lack of practical experience and nuclear medicine or other relevant facilities. Therefore, it would be more than advantageous to research and develop some noninvasive approaches to predict ALNMs preoperatively.

Ultrasound, mammogram, PET/CT, and MRI have been used to diagnose ALNMs during breast cancer staging. Ultrasonography showed a sensitivity and a specificity of 33–86.2% and 40.5–96.2%, respectively [8–13]. The sensitivity and specificity of mammogram procedures were 21% and 99.5%, respectively [11]. The overall sensitivities and specificities of PET/CT were reported to be 20–80% and 88.6–97%, respectively [8–10, 13, 14]. In addition, ultrasonography is convenient but is also dependent on operator experience. Mammogram and PET-CT can result in unnecessary exposure to harmful ionising radiation. Conversely, due to its low inter-observer variability, hardly any radiation, and improved diagnostic contrast, MRI has become a routine noninvasive diagnostic tool.

Machine learning (ML) is a branch of artificial intelligence that includes algorithms that could enhance diagnosis, treatments, and follow-up neuro-oncology visits by analysing enormous complex datasets [15, 16]. In recent years, there have been some studies on the use of ML to predict ALNM in breast cancer patients. The use of ML in predicting ALNM is not dependent on operator experience levels and is more objective with good repeatability. In addition, the diagnostic performance of ML might be further improved. To avoid overfitting and to adequately assess ML performance, proper training should involve k-fold cross-validation or external testing. However, the results so far are far from being consistent even among themselves. What’s more, no meta-analysis has previously been done to assess the use of ML for predicting ALNM. To address this problem, the present meta-analysis pooled all the published studies concerning the diagnostic performance of ML-based MRI in the prediction of ALNM in breast cancer patients.

## Materials and methods

### Literature search and study selection

A search in PubMed, Embase, Web of Science, and the Cochrane Library was performed until 27 December 2020. It used almost all Medical Subject Heading (MeSH) terms available and free keywords for “Machine Learning”, “Transfer Learning”, “Breast Neoplasms”, “Breast Tumor”, “Breast Cancer”, “Lymphatic Metastasis”, and “Lymph Node Metastasis”. A search of the reference lists from included studies was also performed.

Two reviewers selected potentially relevant studies independently based on the title and abstract, and disagreements were resolved by a third reviewer to reach a consensus.

Studies were included if (1) the research subject was limited to human subjects in English; (2) the diagnostic performance pertaining to sensitivity and specificity was reported; (3) a histopathologically confirmed ALNM was present in breast cancer patients; and (4) ML was applied to predict ALNM without defined limit for age or sample size.

Studies were excluded if (1) the publication was on animal research, a conference abstract, or a review article, and (2) the study reported on overlapping patient cohorts.

### Data extraction

Data from the included studies were collected by two investigators independently, and discrepancies between were resolved with the help of a third investigator. Each study was initially identified by identifying the author’s name and the year of publication. A spreadsheet was used to extract total patient populations, numbers of abnormal and normal lymph nodes, and sensitivity and specificity of ML detection. Other information included the study design, algorithms, data sources, MRI sequences, image segmentations, magnet field strengths, and manufacturers.

### Quality assessment

The quality of studies and likelihood of bias were conducted according to the Quality Assessment of Diagnostic Accuracy Studies-2 (QUADAS-2) [17], which has two main areas, viz. risk of bias and concerns regarding applicability. The tool consists of four domains, including patient selection, index test, reference standard, and flow and timing. The first three domains were also assessed in terms of applicability concerns using high, low, or unclear ratings. For individual studies, each domain was considered at a high, low, or unclear risk of bias. If the answers to all signalling questions for a domain were “yes”, the risk of bias would be judged as low. If answers to any signalling questions were “no or unclear”, the risk of bias would be judged as high or unclear. Two reviewers performed quality assessments independently and discrepancies between them were resolved by a third reviewer to reach a consensus. Review Manager 5.3 (RevMan 5.3) software (Cochrane Collaboration, Oxford, UK) was used.

### Statistical analysis

Numerical values for sensitivity and specificity were extracted and their true positive (TP), false positive (FP), false negative (FN), and true negative (TN) values were recalculated. Threshold analysis was performed, and a Spearman correlation coefficient and p value were obtained. Symmetry or asymmetry summary receiver operating characteristic (SROC) curves were used to evaluate threshold effects according to the *p* value of the *b* coefficient using measures of effectiveness (MOEs) modelling. Cochran-Q tests and the inconsistency indices (*I*^2^) of the sensitivity, specificity, positive likelihood ratio (PLR), negative likelihood ratio (NLR), negative predictive value(NPV), and diagnostic odds ratio (DOR) were used to explore heterogeneity. If *I*^2^ < 50% and *p* > 0.05, the fixed-effects model was used; otherwise, the random-effects model was used to pool these five effect sizes. The criteria of heterogeneity for the *I*^2^ values were 0–25% (very low), 25–50% (low), 50–75% (medium), and > 75% (high), respectively. Subgroup analysis was further performed to explore the sources of heterogeneity that were performed based on MRI sequences, magnet field strengths, image segmentation methods, and ML algorithms. Sensitivity analysis was used to assess the robustness of the meta-analysis by verifying if the size of a research study can affect the pooled results. Deeks funnel plot was used to assess publication bias. Data analysis was performed using Stata14.0 (StataCorp LP, College Station, TX) and MetaDisc1.4 (http://www.hrc.es/investigacion/metadisc_en.htm) software. For each of the parameters (1.5 T; 3.0 T; T2-FS; T1CE; DWI; SVM; LR; LDA; 2D and 3D), we constructed forest plots for pooled sensitivities, specificities, PLR, NLR, and DOR. Others such as field strength, sequence, algorithm, and segmentation were compared by using a Student t test, Mann–Whitney U test, or a one-way analysis of variance. For studies describing different results in a classifier due to multiple kernels in ML, performance of these studies was selected with the highest one.

## Results

### Literature search and data extraction

**Fig. 1:**
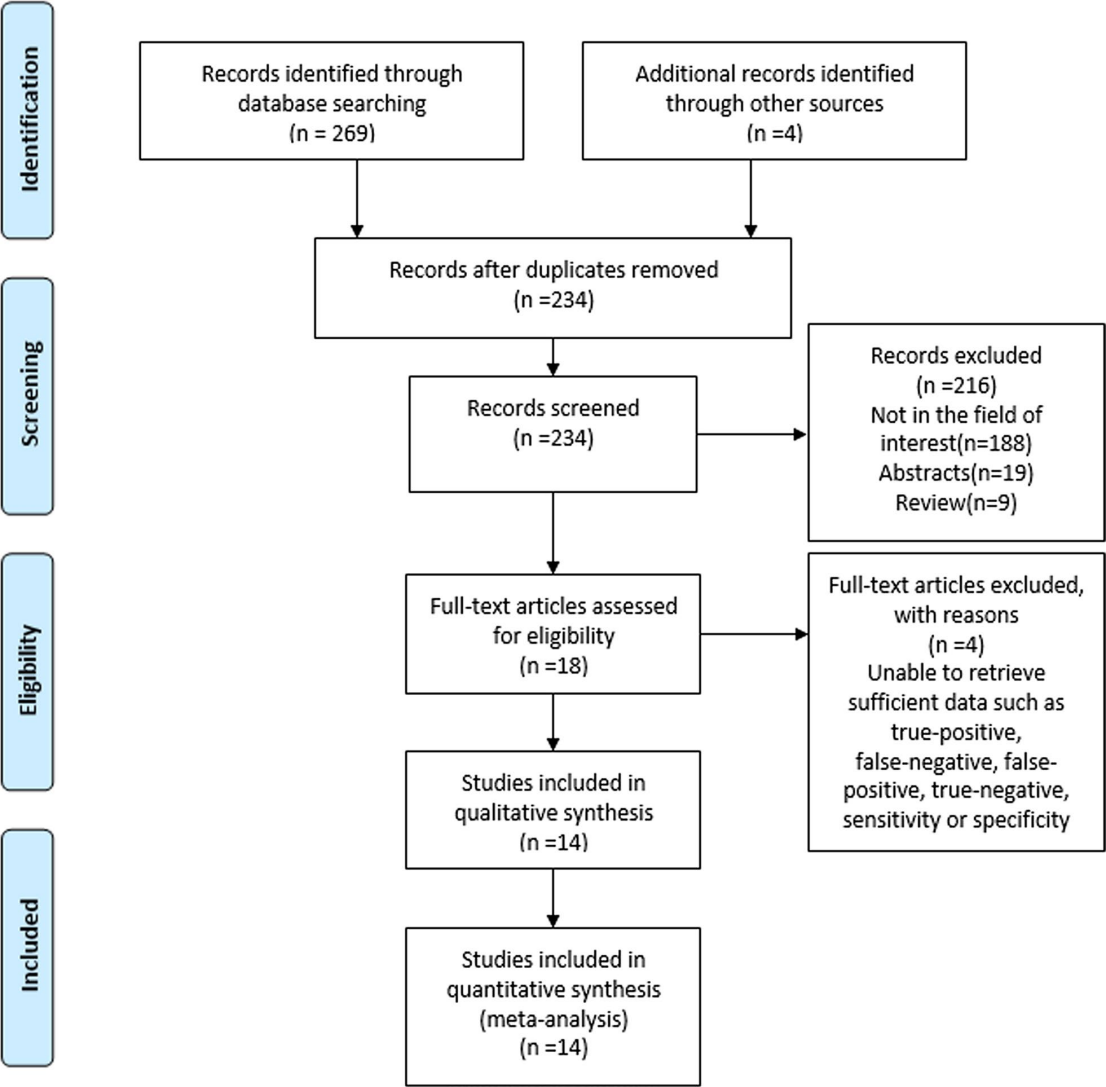
A flow diagram of the literature review and study selection

A detailed study selection process is presented in Fig. 1. There were 273 potentially eligible citations. After removing 39 duplicate records, 234 records were screened. With 216 citations based on the title and abstract excluded, 18 full-text articles were assessed for eligibility. After revision, a total of 14 original articles [18–31] that included 2247 breast cancer patients (954 abnormal lymph nodes and 1508 normal lymph nodes) were eventually included in the study. The patient and study characteristics are described in Table 1, and the baseline characteristics are shown in Table 2.

**Table 1:** Study and patient characteristics

Author (year of publication)	Data source	# Abnormal/normal nodes	# Patients	Magnet field strength, manufacturer	Study design
Hongna Tan (2020)	Single institution	119/210	329	3.0 T GE	Retro
Thomas Ren (2020)	Single institution	66/193	99	1.5 T GE	Retro
Xiao Zhang (2019)	Single institution	55/91	146	1.5 T Philips	Retro
Jia Liu (2019)	Single institution	35/27	62	3.0 T GE	Retro
Karl D Spuhler (2019)	Single institution	55/108	163	1.5 T GE	Retro
Lu Han (2019)	Single institution	148/263	411	1.5 T GE	Retro
Jiaxiu Luo (2018)	Single institution	74/98	172	1.5 T Philips	Retro
Chunling Liu (2019)	Single institution	55/108	163	1.5 T GE	Retro
Yuhao Dong (2018)	Single institution	55/91	146	1.5 T Philips	Retro
Meijie Liu (2020)	Single institution	78/86	164	3.0 T GE	Pros
Xiaoyu Cui (2019)	Single institution	52/63	102	3.0 T Siemens	Retro
Demircioglu (2020)	Single institution	34/50	84	1.5 T Siemens	Retro
Arefan (2020)	Single institution	80/74	154	3.0 T Siemens	Retro
Fusco (2018)	Single institution	48/46	52	1.5 T Aurora	Retro

Retro, retrospective; Pros, prospective

**Table 2:** Baseline characteristics of the included studies

Author (year of publication)	Algorithm	Sequences	Segmentation	Dataset	Sensitivity	Specificity
Hongna Tan (2020)	SVM	T2-FS	3D	Training set	84.38%	72.25%
				Validation set	65.22%	81.08%
Thomas Ren (2020)	CNN	T1CE	2D	Validation set	92.10 ± 2.90%	79.30 ± 5.10%
Xiao Zhang (2019)	RF	DWI	3D	Validation set	83.30%	74.20%
		T2-FS			77.80%	87.10%
Jia Liu (2019)	SVM	T1CE	3D	Training set	75.00%	76.00%
				Validation set	71.00%	100.00%
	Xgboost			Training set	89.00%	76.00%
				Validation set	86.00%	83.00%
	LR			Training set	71.00%	71.00%
				Validation set	71.00%	83.00%
Karl D Spuhler (2019)	CNN	T1CE	3D	Testing set	72.20%	88.90%
Lu Han (2019)	SVM	T1CE	2D	Training set	89.00%	57.00%
				Validation set	78.00%	72.00%
Jiaxiu Luo (2018)	SVM	DWI	3D	Testing set	86.70%	83.30%
Chunling Liu (2019)	LR	T1CE	3D	Training set	76.10%	66.70%
				Validation set	81.90%	77.80%
Yuhao Dong (2018)	LR	T2-FS	3D	Training set	66.30 ± 0.30%	81.60 ± 0.20%
				Validation set	60.00 ± 0.60%	74.70 ± 0.40%
		DWI		Training set	74.00 ± 0.20%	80.80 ± 0.20%
				Validation set	69.50 ± 0.50%	75.70 ± 0.40%
		T2-FS and DWI		Training set	66.30 ± 0.30%	81.60 ± 0.20%
				Validation set	70.00 ± 0.80%	74.70 ± 0.50%
Meijie Liu (2020)	LR	T1CE	2D	Validation set	64.00%	79.00%
Xiaoyu Cui (2019)	SVM	T1CE	3D	Validation set	94.90%	77.96%
	KNN				89.39%	87.18%
	LDA				80.31%	67.78%
Demircioglu (2020)	LR	T1CE and T2-FS	3D	Validation set	71.00%	74.00%
Arefan (2020)	LDA	T1CE	2D	Testing set	60.00%	87.00%
	RF				60.00%	86.00%
	NB				81.00%	67.00%
	KNN				74.00%	54.00%
	SVM				70.00%	71.00%
	LDA		3D		63.00%	92.00%
	RF				64.00%	90.00%
	NB				85.00%	62.00%
	KNN				67.00%	58.00%
	SVM				81.00%	50.00%
	LDA		2D	Validation set	82.00%	76.00%
	RF				89.00%	68.00%
	NB				73.00%	82.00%
	KNN				79.00%	75.00%
	SVM				65.00%	88.00%
	LDA		3D		82.00%	78.00%
	RF				89.00%	70.00%
	NB				69.00%	76.00%
	KNN				75.00%	75.00%
	SVM				73.00%	79.00%
Fusco (2018)	LDA	T1CE	3D	Validation set	88.50%	77.80%

CNN, convolutional neural networks; SVM, support vector machine; LR, linear regression; KNN, k-nearest neighbor; LDA, linear discriminant analysis; NB, naive Bayes; RF, random forest; T2-FS, fat-suppressed T2; T1CE, contrast-enhanced T1. The algorithm pooled was chosen with the highest performance

### Data quality assessment

**Fig. 2:**
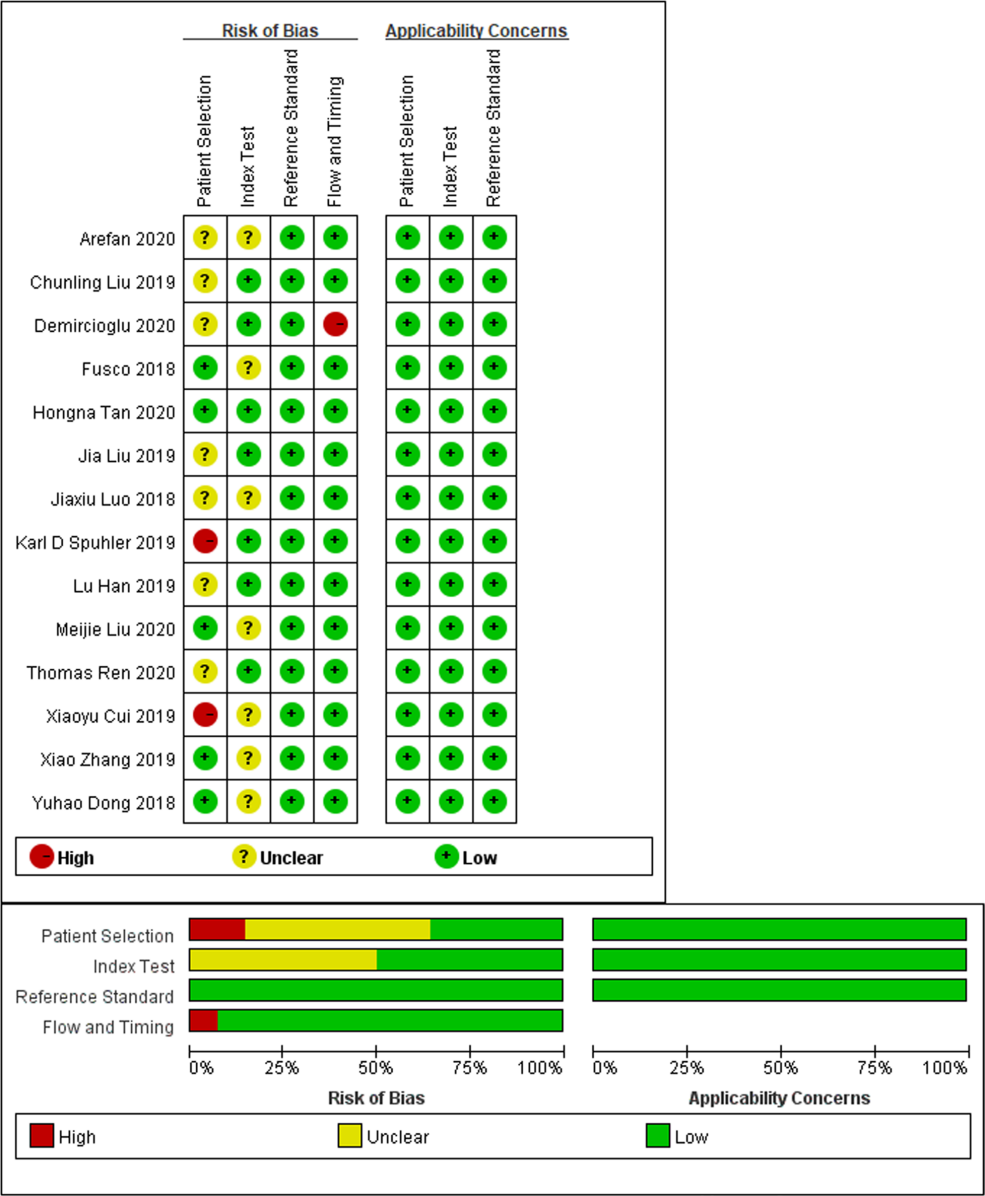
Methodologic quality of the included studies assessed according to the Quality Assessment of Diagnostic Accuracy Study 2 tool for risk of bias and applicability concerns. Green represents low, yellow unclear, and red high risk of bias

Results of the QUADAS-2 assessments are shown in Fig. 2 and the Additional file 1: Supplementary Materials. Most included studies were regarded as having a low to moderate risk of bias with low concerns over their applicability. In particular, only one study scored a low risk of bias in all domains [19]. For the patient selection domain, two studies were considered to have a high risk of bias due to their non-consecutive or random patient enrolment [24, 27]. Seven studies were considered to have a risk that is uncertain because they did not explain how patients were enrolled [20–23, 26, 28, 30]. For the index test domain, seven studies [18, 22, 25, 27, 29–31] were considered to have an unclear risk because they lacked pre-specified thresholds. Further, for the reference standard domain, all studies were classified as having a low risk, and all of the selected studies used biopsy and/or histopathology as the reference standard. Lastly, for the domain of flow and timing, one study [21] was considered to be at high risk because it did not explain whether all of its patients were included in the study.

### Data analysis

**Fig. 3:**
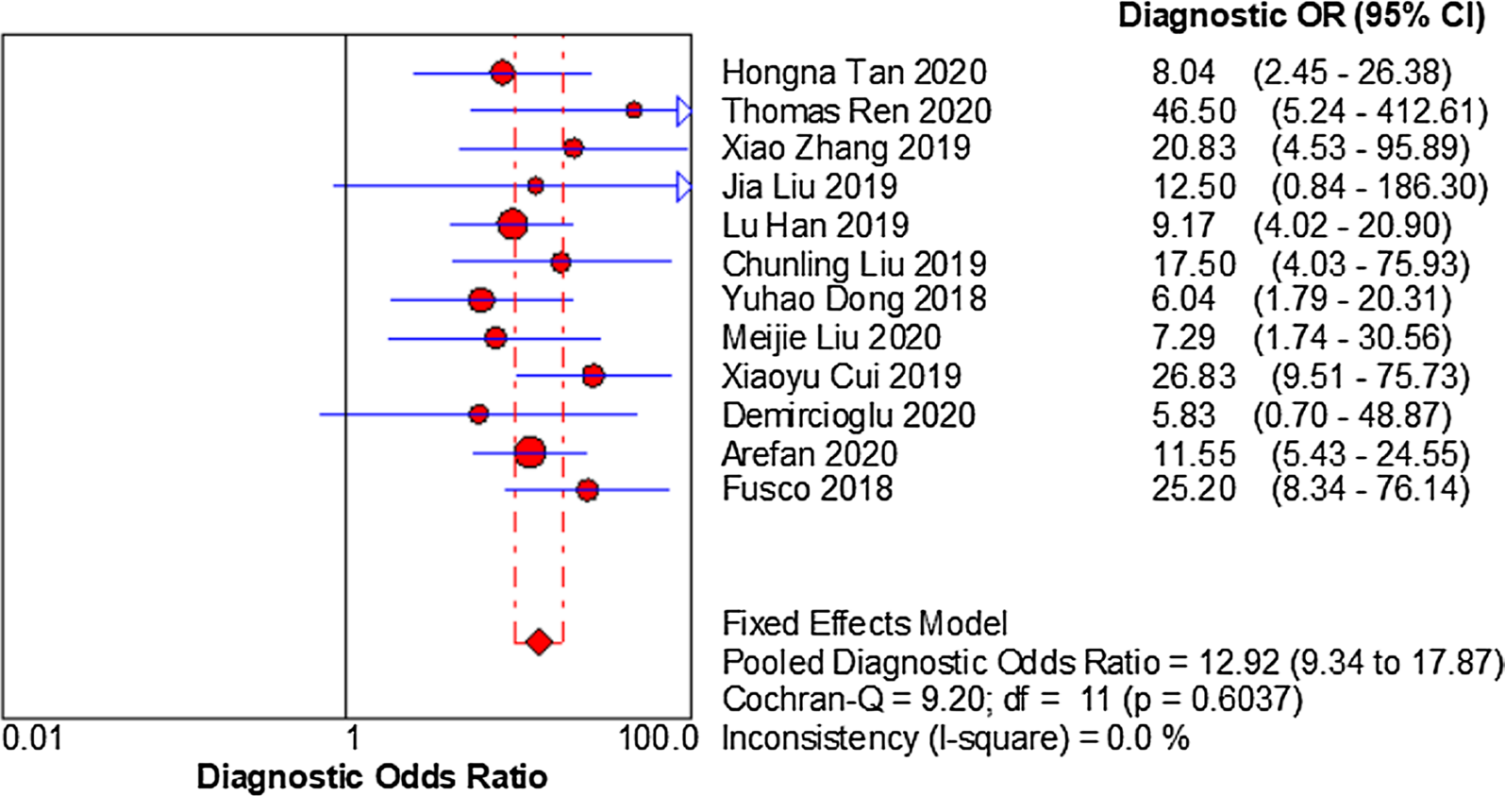
A forest plot of single studies for the pooled diagnostic odds ratio and 95% confidence interval (CI) in the validation group. Horizontal lines represent the 95% CI of the point estimates. Each red circle represents the area under the curve (AUC) of the individual studies, and the box size indicates the study size. The red diamond indicates the pooled AUC of all twelve studies

**Fig. 4:**
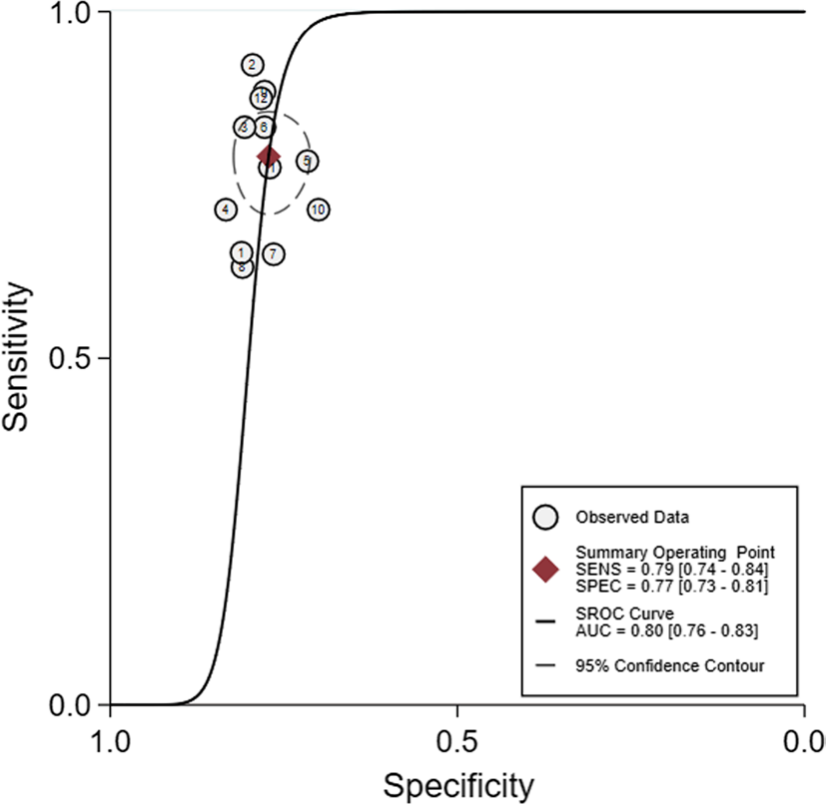
Summary receiver operating characteristics (SROC) curve regarding the diagnostic performance of machine learning in predicting axillary lymph node metastasis in breast cancer patients. SROC curve (solid line) and summary point (red square). Every circle represents the sensitivity and specificity estimate from one study, and the size of the circle reflects the sample size. The pooled area under the curve was 0.80

**Fig. 5:**
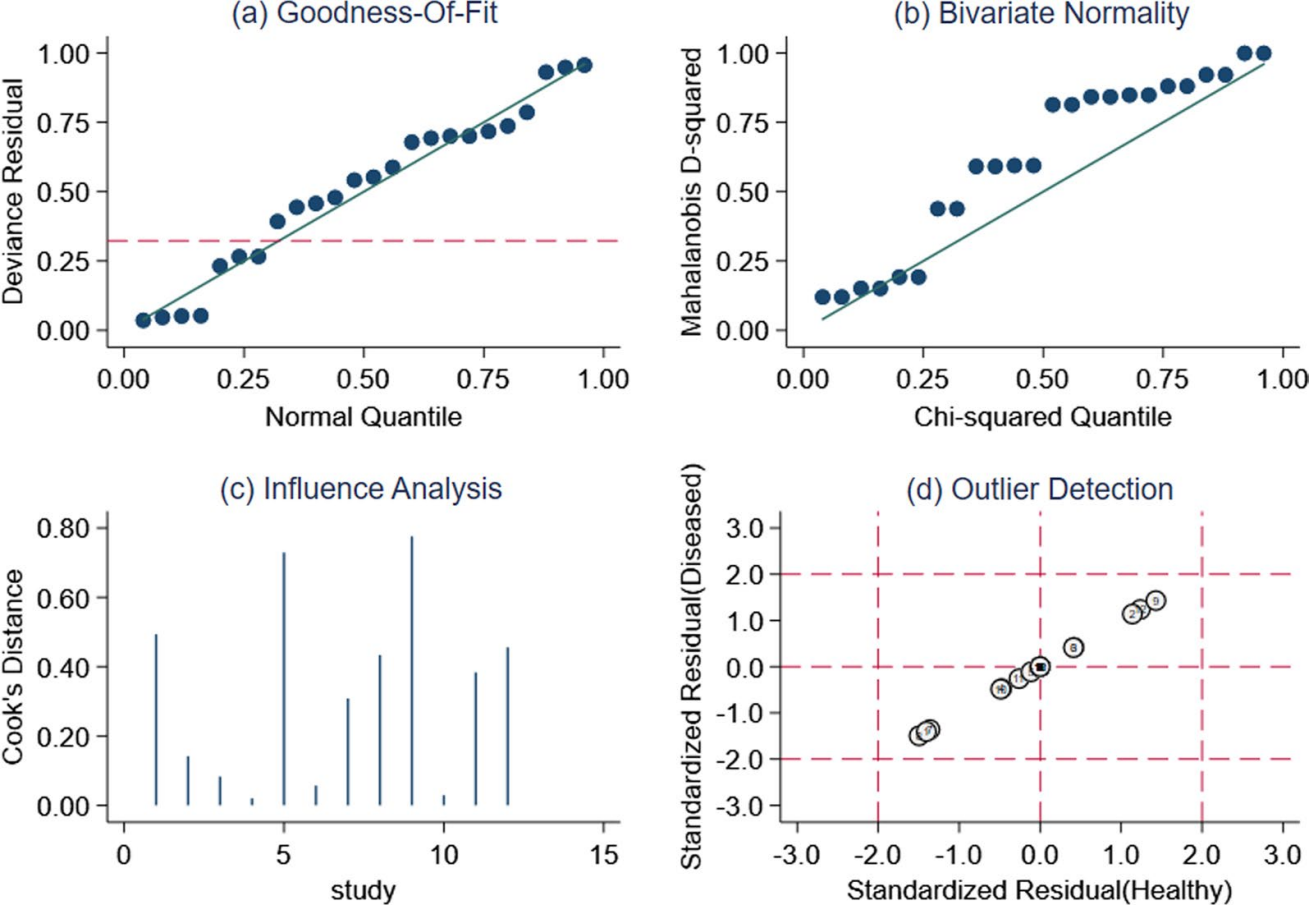
Sensitivity analysis. **a** Goodness of fit; **b** bivariate normality; **c** influence analysis; and **d** outlier detection. All original articles had high stability and reliability and relatively high quality in the validation set

There are different numbers for training, testing, and validation studies because there were eight studies which only showed the validation or test results [18, 20, 21, 24, 25, 27, 29, 30], whereas six others only showed the results of training, testing/validation [19, 22, 23, 26, 28, 31]. In the test set, the sensitivity and specificity of the pooled three studies were 76% and 82%, respectively. In the validation set, the sensitivity and specificity of the pooled twelve studies were 79% and 77%, respectively. The Spearman correlation coefficient in the validation set was − 0.083 (*p* = 0.799), indicating no threshold effect discovered. Next, no “shoulder arm” plot was observed in the SROC curve. Since *I*^2^ of sensitivity, specificity, PLR, NLR, DOR = 21.60%, 0.00%, 0.00%, 24.20%, 0.00% < 50.00%, *p* values were 0.174, 0.994, 0.969, 0.206, 0.604, respectively, a model of fixed-effects to pool effect sizes was chosen (Fig. 3). The pooled sensitivity, specificity, PLR, NLR, and DOR were 0.79 (95%Cl 0.74–0.84), 0.77 (95%Cl 0.73–0.81), 3.47 (95%Cl 2.91–4.14), 0.27 (95%Cl 0.22–0.33), 12.92 (95%Cl 9.34–17.87), respectively (Fig. 4). The AUC was 0.80(95%Cl 0.76–0.81). More interestingly, for those patients predicted to have negative SLN, they achieved a high NPV of 0.83. Sensitivity analysis that removed studies with potential bias showed results consistent with the primary meta-analysis, which were conducted to assess robustness of the synthesised results. Sensitivity analysis showed that twelve original articles had better stability and reliability and relatively high quality (Fig. 5).

**Fig. 6:**
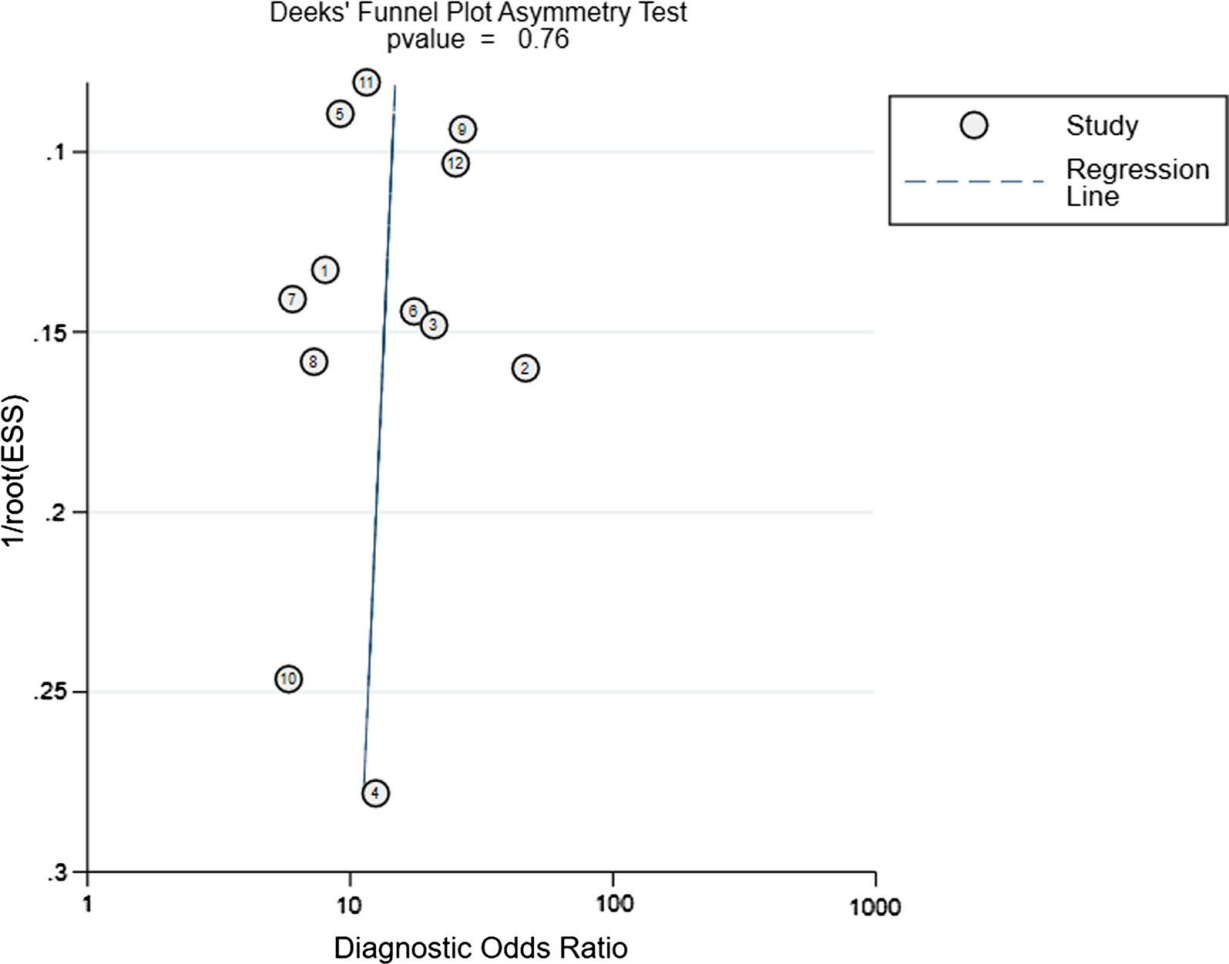
The Deeks funnel plot asymmetry test to determine publication bias in the literature evaluation (*p* = 0.76) indicated there was no obvious publication bias. Each dot represents an individual study

Since the studies of the test set were too small to draw any reliable conclusions, subgroup analysis was performed only in the validation set (Table 3). T1CE with ML yielded a higher sensitivity (0.80 vs. 0.67 vs. 0.768) than T2-FS and DWI (*p* < 0.05). The number of combining T2-FS and DWI or T2-FS and T1CE was only one study, which, nonetheless, was too small to draw any reliable conclusions. In addition, MRI magnet field strength also affected the diagnostic performance of ML. In comparison with 1.5 T, studies using 3.0 T had a better sensitivity (0.86 vs. 0.81) (*p* > 0.05). In algorithm, SVM demonstrated a higher specificity than LR and LDA (0.79 vs. 0.78 vs. 0.75), whereas LDA showed a higher sensitivity than LR and SVM (0.83 vs. 0.70 vs.0.77) (*p* < 0.05). ML performed better for 3D than 2D, in which the pooled sensitivities were 0.80 versus 0.77 and specificities were 0.78 versus 0.76 (*p* > 0.05). In addition, the Deeks funnel plot revealed that there was no obvious publication bias. (*p* = 0.870, Fig. 6).

**Table 3:** The performance evaluation of the datasets and subgroups

Dataset	No. of studies	Sensitivity	Specificity	PLR	NLR	DOR
Testing set	3	0.76 (0.64–0.86)^#^	0.82 (0.72–0.89)^##^	3.90 (2.49–6.11)^##^	0.29 (0.19–0.46)^#^	12.88 (5.99–27.71)^##^
Validation set	12	0.79 (0.74–0.84)^#^	0.77 (0.73–0.81)^#^	3.47 (2.91–4.14)^#^	0.27 (0.22–0.33)^#^	12.92 (9.34–17.87)^#^
Overall validation group						
*Magnetic field strength (T)*						
1.5 T	7	0.81 (0.75–0.87)^#^	0.76 (0.71–0.81)^#^	3.38 (2.70–4.24)^#^	0.25 (0.18–0.34)^#^	13.15 (8.37–20.66)^#^
3.0 T	5	0.86 (0.78–0.89)^**^	0.76 (0.70–0.82)^#^	3.46 (2.69–4.46)^#^	0.23 (0.11–0.48)^**^	17.78 (10.54–30.00)^##^
*Sequence*						
T2-FS	3	0.67 (0.54–0.79)^#^	0.80 (0.71–0.88)^#^	3.43 (2.22–5.29)^##^	0.41 (0.28–0.59)^#^	8.01 (4.01–16.00)^##^
T1CE	8	0.80 (0.75–0.84)^##^	0.80 (0.75–0.84)^#^	4.04 (3.25–5.02)^#^	0.25 (0.19–0.31)^##^	16.27 (11.08–23.89)^#^
DWI	2	0.76 (0.60–0.89)^#^	0.75 (0.63–0.85)^#^	3.10 (1.96–4.91)^#^	0.31 (0.17–0.56)^#^	10.00 (3.97–25.17)^#^
*Algorithm*						
SVM	5	0.77 (0.71–0.82)^**^	0.79 (0.73–0.83)^##^	3.67 (2.87–4.70)^#^	0.30 (0.19–0.47)^*^	13.43 (8.62–20.93)^##^
LR	4	0.70 (0.58–0.81)^#^	0.78 (0.68–0.85)^#^	3.20 (2.17–4.71)^#^	0.37 (0.26–0.55)^#^	8.60 (4.44–16.66)^#^
LDA	3	0.83 (0.77–0.88)^#^	0.75 (0.68–0.81)^#^	3.35 (2.58–4.35)^##^	0.22 (0.16–0.31)^#^	14.78 (9.01–24.26)^#^
*Segmentation*						
2D	4	0.77 (0.70–0.83)^##^	0.76 (0.70–0.82)^#^	3.30 (2.55–4.28)^#^	0.29 (0.21–0.39)^#^	11.46 (7.05–18.62)^#^
3D	9	0.80 (0.75–0.84)^##^	0.78 (0.73–0.82)^#^	3.56 (2.88–4.39)^#^	0.26 (0.21–0.34)^##^	13.52 (9.27–19.72)^#^

PLR, positive likelihood ratio; NLR, negative likelihood ratio; DOR, diagnostic odds ratio; SVM, support vector machine; LR, linear regression; LDA, linear discriminant analysis; T2-FS, fat-suppressed T2; T1CE, contrast-enhanced T1; #, 0–25% of I2 values; ##, 25–50%; *, 50–75%; **, > 75%; # and ##, fixed-effects model; * and **, random-effects model

## Discussion

ALNM can determine the prognosis and treatment of breast cancer patients. Thus, it is imperative to find an accurate and reproducible way to detect ALNM. To address this issue, this meta-analysis was intended to assess the applicability of ML to the classification of ALNM on MRI and to the identification of potential covariates that influence the diagnostic performance of ML.

DWI is a commonly performed sequence with promising results for the evaluation of breast lesions because their images can be obtained in a short time without contrast agents [32]. However, most lesions show images with a relatively inferior quality and increase blurrings and distortions on DWI, which can make it difficult to accurately segment the lesions. In contrast, T2-weighted imaging (T2WI) can clearly depict edema, hemorrhage, mucus, and cystic fluid, which can be valuable for evaluation of breast masses [33]. T2-FS can also show the lesion boundaries clearly. Dynamic contrast-enhanced (DCE)-MRI with numerous scanning phases is sensitive to the change in tissue vessel perfusion and permeability [34]. However, compared with T2-FS, DCE-MRI had a better sensitivity. Demircioglu et al. [21] reported that ALNM classifications that combined T2-FS with T1CE were moderate with an AUC of 0.710, but this meta-analysis discovered that the number of combined sequences was not big enough to draw any reliable conclusions from them. Ren et al. [20], Han et al. [26] and Liu et al. [28] used the T1CE first axial phase images to predict ALNM and achieved AUCs of 0.91, 0.78, and 0.81 in the validation sets, respectively. Liu et al. [23], Liu et al. [18], Arefan et al. [22], Fusco et al. [29], and Cui et al. [27] use the peak enhanced phase images achieved AUCs of 0.85, 0.74, 0.82, 0.81, and 0.77 in the validation sets, respectively. Theoretically, 3 T imaging has been shown to be able to improve image quality due to its better performance resulting from higher spatial resolution [35]. This meta-analysis revealed that, in comparison with 1.5 T, studies using 3.0 T had a better sensitivity (0.86 vs. 0.81), albeit without significant differences. This should be further validated with a larger size of samples in the future.

Different as they are, algorithms have advantages of their own. Linear discriminant analysis (LDA) models can distinguish and identify a linear decision boundary between classes [36]. Support vector machine (SVM), as one of the most popular classifying techniques, is an excellent algorithm that can be utilised to model misspecifications and can effectively handle high-dimensional data [37]. Linear regression (LR) is also suitable for the regression of high-dimensional data. In this meta-analysis, SVM demonstrated a higher specificity than LR and LDA, whereas LDA showed a higher sensitivity than LR and SVM. Yu et al. [38] used LR to identify ALNM in the development and validation set with AUCs of 0.88 and 0.85, respectively. Takada et al. [39] used a decision tree to predict ALNM (AUCs of 0.770 and 0.772 in the training and validation sets, respectively). Schacht et al. [40] and Ha et al. [41] used neural net classifiers to predict ALNM, achieving an AUC of 0.880 and an accuracy of 84.30%. As probabilistic classifiers, naive Bayes models are constructed using the Bayes theorem of conditional probabilities [42]. XGBoost is an optimised distributed gradient boosting library designed to be highly efficient, flexible, and portable. A random forest is a multitude of trees, but they are differentiated by a random selection of the variables to reduce correlations between the fitted trees [43]. The k-nearest neighbor algorithm is a tool used to exploit the local information in classification and regression problems [44]. Convolutional neural networks (CNNs) can obtain global and local image information directly from the convolution kernels [45, 46]. However, the data of these algorithms were insufficient. Multiple ML models should be used in clinical routine in order that the ensemble of models has a better diagnostic performance than individual one.

The 2D image segmentation method, using a single tumour slice, can make 2D analysis susceptible to the choices of the single representative slice chosen by human experts, whereas 3D analysis is not susceptible to this variation because they cover all tumour volumes. Although it is exceedingly time-consuming to perform manual segmentation using 3D imaging, most tumour characteristics can be captured with 3D tumour volumes. Thus, this may indicate why 3D performs better than 2D, albeit we did not find any significant differences.

The results of this meta-analysis were encouraging owing to its pooled sensitivity of around 0.80, which, however, means that 1 in 5 women that would go with undetected metastases and this may have a detrimental effect on overall survival for 20% of patients with positive SLN status. Although a high NPV of 0.83 means that ML may potentially benefit those with negative SLN, who account for over 70% of all breast cancer patients [7], by helping them eliminate the unnecessary invasive lymph node removal and avoid the overtreatment of axillary fossa accompanied by the associated serious complications, this would translate to 1 in 5 tests being false negative. In view of this, we would like to admit that ML may not be yet usable in routine clinical checkups especially when using patient survival as a primary measurement of outcome.

Several limitations to our meta-analysis are noticeable. First, only fourteen original research articles met the selection criteria as there were not many studies about ALNM in breast cancer patients. We were also unable to retrieve sufficient data for some studies. Second, the algorithm classification performances varied from feature selection to alterations in the linear, quadratic, cubic, and Gaussian kernel functions; therefore, we chose the highest performers that might have affected the performance results. Finally, in all cases, the use of data was from only one single institution, which may not be sufficient to demonstrate the replicability of our findings.

In conclusion, MRI sequences and algorithms constitute the main factors that affect the diagnostic performance of machine learning. Combining SLNB with ML, it would be more helpful to breast cancer patients. In future research, larger, well-designed, conducted, and reported trials are needed for better performances.

## Supplementary Information

**Additional file 1**. Detailed data quality assessment.

## Data Availability

All data generated or analysed during this study are included in this article.

## Abbreviations

ALNM: Axillary lymph node metastasis
AUC: Area under the receiver operating characteristic curve
CI: Confidence interval
CNN: Convolutional neural network
DOR: Diagnostic odds ratio
LDA: Linear discriminant analysis
LR: Linear regression
ML: Machine learning
NLR: Negative likelihood ratio
PLR: Positive likelihood ratio
QUADAS-2: Quality Assessment of Diagnostic Accuracy Studies-2
SROC: Summary receiver operating characteristic
SVM: Support vector machines
T1CE: Contrast-enhanced T1
T2-FS: Fat-suppressed T2
