# Supplementary material for: A meta-analysis of the diagnostic performance of machine learning-based MRI in the prediction of axillary lymph node metastasis in breast cancer patients
Source: Insights Imaging. 2021 Nov 3;12:156. doi: 10.1186/s13244-021-01034-1 (PMC8566689; doi:10.1186/s13244-021-01034-1)
Supplement: Supplementary file 1 — Additional file 1. Detailed data quality assessment. [file 13244_2021_1034_MOESM1_ESM.docx]

**ELECTRONIC SUPPLEMENTARY MATERIAL**

***Literature search***

A detailed study selection process is presented in Fig. 1. There were 273 potentially eligible citations. After removing 39 duplicate records, 234 records were screened. After screening the titles and abstracts, 18 articles remained, of which 216 articles were further excluded for the following reasons: outside the topic of interest (n = 188), review article (n = 9), and conference article (n = 19). Next, full-text reviews of 14 potentially eligible articles were performed, and 4 articles were excluded for insufficient data. Finally, a total of fourteen original articles^[1-14]^ including 2261 patients were included and analysed in this study.

***Data extraction and quality assessment***

Thirteen of the selected studies had a retrospective design and one study had a prospective design. Nine of them used contrast enhanced T1(T1CE) ^[1, 3, 5-7, 9-12]^; two of them used fat-suppressed T2(T2-FS) and DWI^[8, 14]^; one of them used only T2-FS^[2]^; one of them used only DWI^[13]^; one of them used T2-FS and T1CE^[4]^;

Four of them used linear regression(LR)^[1, 4, 11, 14]^; three of them used support vector machine(SVM)^[2, 9, 13]^; two of them used convolutional neural networks^[3, 7]^; one of them used only random forest(RF)^[8]^; one of them used only linear discriminant analysis(LDA)^[12]^; one of them used SVM, Xgboost and LR^[6]^; one of them used SVM, k-Nearest Neighbor (KNN) and LDA^[10]^; one of them used SVM, KNN, LDA, RF and Naive Bayes^[5]^;

Six studies included training set and validation set^[2, 5, 6, 9, 11, 14]^; six studies included only validation set^[1, 3, 4, 8, 10, 12]^; two studies included only testing set^[7, 13]^;

The QUADAS-2 scores are shown in Fig. 2 and supplementary materials. Most included studies were regarded as having a low to moderate risk of bias with low concerns regarding applicability. In particular, only one study scored a low risk of bias in the all domains^[2]^. For the patient selection domain, two studies were considered to show a high risk of bias due to non-consecutive or random patient enrolment^[7, 10]^ and seven studies were considered as having an unclear risk since they did not clarify how patients were enrolled^[1-4, 7, 9, 11]^. For the index test domain, seven studieswere considered to have an unclear risk^[1, 5, 8, 10, 12-14]^, because they lacked pre-specified thresholds. Further, for the reference standard domain, all studies were classified as having a low risk and all of the selected studies used biopsy and/or histopathology as the reference standard. Lastly, for the domain of flow and timing, one studie was considered to show a high risk because it did not clarify whether all the patients were included in the studies^[4]^.

**Reference**

1. Liu M, Mao N, Ma H, et al(2020) Pharmacokinetic parameters and radiomics model based on dynamic contrast enhanced MRI for the preoperative prediction of sentinel lymph node metastasis in breast cancer. Cancer Imaging 20(1)

2. Tan H, Gan F, Wu Y, et al(2020) Preoperative Prediction of Axillary Lymph Node Metastasis in Breast Carcinoma Using Radiomics Features Based on the Fat-Suppressed T2 Sequence. Academic Radiology 27(9):1217-1225

3. Ren T, Cattell R, Duanmu H, et al(2020) Convolutional Neural Network Detection of Axillary Lymph Node Metastasis Using Standard Clinical Breast MRI. Clinical Breast Cancer 20(3):e301-e308

4. Demircioglu A, Grueneisen J, Ingenwerth M, et al(2020) A rapid volume of interest-based approach of radiomics analysis of breast MRI for tumor decoding and phenotyping of breast cancer. PLoS One 15(6):e234871

5. Arefan D, Chai R, Sun M, et al(2020) Machine learning prediction of axillary lymph node metastasis in breast cancer: 2D versus 3D radiomic features. MEDICAL PHYSICS

6. Liu J, Sun D, Chen L, et al(2019) Radiomics analysis of dynamic contrast-enhanced magnetic resonance imaging for the prediction of sentinel lymph node metastasis in breast cancer. Frontiers in Oncology 9(SEP)

7. Spuhler K D, Ding J, Liu C, et al(2019) Task-based assessment of a convolutional neural network for segmenting breast lesions for radiomic analysis. Magnetic Resonance in Medicine 82(2):786-795

8. Zhang X, Zhong L, Zhang B, et al(2019) The effects of volume of interest delineation on MRI-based radiomics analysis: evaluation with two disease groups. Cancer Imaging 19(1):89

9. Han L, Zhu Y, Liu Z, et al(2019) Radiomic nomogram for prediction of axillary lymph node metastasis in breast cancer. Eur Radiol 29(7):3820-3829

10. Cui X, Wang N, Zhao Y, et al(2019) Preoperative Prediction of Axillary Lymph Node Metastasis in Breast Cancer using Radiomics Features of DCE-MRI. Sci Rep 9(1):2240

11. Liu C, Ding J, Spuhler K, et al(2019) Preoperative prediction of sentinel lymph node metastasis in breast cancer by radiomic signatures from dynamic contrast-enhanced MRI. J MagnReson Imaging 49(1):131-140

12. Fusco R, Sansone M, Granata V, et al(2018) Use of Quantitative Morphological and Functional Features for Assessment of Axillary Lymph Node in Breast Dynamic Contrast-Enhanced Magnetic Resonance Imaging. BioMed Research International

13. Luo J, Ning Z, Zhang S, et al(2018) Bag of deep features for preoperative prediction of sentinel lymph node metastasis in breast cancer. Phys Med Biol 63(24):245014

14. Dong Y, Feng Q, Yang W, et al(2018) Preoperative prediction of sentinel lymph node metastasis in breast cancer based on radiomics of T2-weighted fat-suppression and diffusion-weighted MRI. Eur Radiol 28(2):582-591

Table 4 Summary of QUADAS-2 tool assessment for all reviewed studies

+, high; -, low; ?, unclear
